# Vitamin D Knowledge, Attitudes, and Behaviors in Young Danish Women with a Non-Western Ethnic Minority Background—A Questionnaire Survey

**DOI:** 10.3390/ijerph17218053

**Published:** 2020-11-01

**Authors:** Erdinc Özel, Lourdes Cantarero-Arevalo, Ramune Jacobsen

**Affiliations:** 1 Department of Pharmacy, University of Copenhagen, 2100 Copenhagen, Denmark; dbf339@alumni.ku.dk (E.Ö.); lou.cantarero@sund.ku.dk (L.C.-A.); 2 WHO Collaborating Center for Research and Training in Patient Perspective on Medicines Use, Department of Pharmacy, University of Copenhagen, 2100 Copenhagen, Denmark

**Keywords:** vitamin D, ethnicity, knowledge, Denmark

## Abstract

The prevalence of vitamin D deficiency in women with a non-Western ethnic minority background in Nordic countries is high. The aim of this study was to assess vitamin D knowledge, attitudes, and behaviors in women with a non-Western ethic minority background living in Denmark. A validated vitamin D knowledge, attitudes, and behaviors’ questionnaire was translated into Danish, piloted, and distributed via relevant Facebook groups. The responses were analyzed using parametric and non-parametric tests for descriptive and bivariate analyses. In total, 254 women who considered themselves having a non-Western ethnic minority background responded to the questionnaire. The median age (IQR) was 25 (23–33) years old; 32% had a professional bachelor’s, 28% had high school, and 22% had a master’s or higher university education. Participants scored higher on vitamin D general knowledge (scores above 80 on the scale 0–100) compared to vitamin D nutrition knowledge or vitamin D attitudes and behaviors (scores around 60 on the scale 0–100). In conclusion, the vitamin D knowledge among study participants—i.e., young well-educated non-Western ethnic minority women in Denmark—was pretty good. The further examination of vitamin D knowledge, attitudes, and behaviors should explore specifics related to nationality and religion and focus on less-educated non-Western ethnic minority women in Denmark and other Nordic countries.

## 1. Introduction

Observational and intervention studies suggest that vitamin D deficiency may increase the risk of developing nutritional rickets, as well as metabolic, autoimmune, infectious, cardiovascular, mental, and other types of disorders [1,2]. Vitamin D deficiency, however, is a recognized public health problem, especially in Nordic countries [3,4]. Non-Western ethnic minorities in Nordic countries exhibit especially high vitamin D deficiency rates [5,6]. Vitamin D deficiency in young non-Western ethnic minority women raises special concerns, as the vitamin D status in young women determines health outcomes not only for the women themselves but also for their potential offspring [7].

A debate, however, exists regarding whether vitamin D optimal levels are naturally lower in darker-skinned populations. A body of evidence points towards generally lower vitamin D levels in dark-skinned human populations, even those receiving intense sunlight. For example, the vitamin D levels were below 75 nmol/L in 51% of young tanned Hawaiians with 22.4 h per week of unprotected sun exposure [8]; among south Indians, “agricultural workers starting their day at 0800 and working outdoors until 1700 with their face, chest, back, legs, arms, and forearms exposed to sunlight”, 44% of the men and 70% of the women had less than 50 nmol/L [9]; in Saudi Arabia, levels were below 25 nmol/L in 35%, 45%, 53%, and 50% of normal male university students of Saudi, Jordanian, Egyptian, and other origins, respectively [10]; in a sample of healthy Middle Eastern athletes, 91% had less than 50 nmol/L [11]. A meta-analysis concluded that vitamin D levels were consistently and significantly lower in people of non-European origin than in people of European origin regardless of latitude [12]. Some variations were also shown among Europeans, with lower vitamin D levels in central and southern Europeans than in lighter-skinned Swedes [13]. Some studies have provided evolutionary explanations [14] and argued for genetic differences [15] in human population causing different vitamin D metabolism (e.g., receptors that bind more tightly to the vitamin D molecule) and thus different vitamin D requirements.

Migration from sunny non-European countries into the north of Europe in any case diminishes exposure to sunshine and negatively affects vitamin D synthesis in the skin. The Danish health authorities therefore recommend vitamin D supplementation for immigrants with darker skin and a sunlight-avoidant lifestyle [6]. The Danish health authorities furthermore are initiating educational campaigns to raise vitamin D deficiency awareness and motivate taking vitamin D supplements for population groups with a non-Western ethnic minority background. For example, in April 2019, the Danish National Board of Health started the campaign called “Do you get enough vitamin D?”, targeted at some non-Western ethnic minority populations [16]. This campaign consisted of the production of educational material about vitamin D published in Turkish, Arabic, and Urdu. As there have been no studies on vitamin D knowledge in non-Western ethnic minority groups in Denmark or other Nordic countries so far, the aim of this study was to explore vitamin D knowledge, attitudes, and behaviors in Danish women with a non-Western ethnic minority background. We also aimed to assess associations between knowledge, attitudes, and behaviors and women’s age, education, and employment.

## 2. Materials and Methods

### 2.1. Questionnaire

To assess vitamin D knowledge, attitudes, and behaviors, a validated questionnaire identified in the literature was used [17]. The questionnaire was translated into Danish and further piloted with five female students who had a non-Western ethnic minority background. During the pilot, three items (Nr 8: “Currently, vitamin D deficiency is one of the most important health issues in our country”; Nr 17: “Urbanization prevents sun exposure and production of required vitamin D”; and 21: “The undesirable taste of sea foods for Iranians is one of the barriers to their consumption of dietary sources of vitamin D”) were considered specific to Iranian background and omitted from the Danish version of the questionnaire. Due to the specifics of the scale scoring system (i.e., recalculating the final scores into 0–100 points), the exclusion of separate items from the scale was possible. The final questionnaire consisted of 10 items for vitamin D general knowledge, 5 items for vitamin D nutrition knowledge, 10 items for vitamin D attitudes, and 10 items for vitamin D behaviors. Vitamin D general and nutrition knowledge questions/items had the response options “Yes”, “No”, and “Don’t Know”, which, depending on the item, gave 2, 0, or 1 points; the points were summed into “general knowledge” and “nutrition knowledge” scales. The vitamin D attitudes and behaviors’ questions/items had five-point Likert scale responses ranging from “strongly disagree” to “strongly agree” for the attitudes; from “never” to “always” for the behaviors’ questions; and, depending on the question, giving 1 to 5 points, which were summed into “attitudes” and “behaviors” scales. All of the above scales were transformed into 0–100-point scores. The higher the score, the better the vitamin D knowledge, attitudes, or behaviors. The difference between the attitudes’ and behaviors’ concepts was that the attitude questions concerned knowledge about the behaviors needed to maintain optimal vitamin D levels (e.g., Nr. 19 “Full time indoor occupation prevents the sun exposure required for production of vitamin D”) while the behavior questions concerned individual’s actual behavior (e.g., Nr. 36 “During the day I am directly exposed to sunlight outdoors”).

To categorize ethnic background, the respondents were asked which ethnicity they identify themselves as: Danish, Western immigrants or descendants, or non-Western immigrants or descendants. Such ethnic background categorization is used by Statistics Denmark based on the information which country a person and her/his parents are born in or immigrated from. In our questionnaire, the respondents were asked to both identify their ethnic category themselves and write down the country they and both of their parents were born in. Statistics Denmark has developed the country grouping “western / non-western countries” and has used it since 2002. According to this grouping, “non-western countries” include Turkey and countries outside Europe except the United States, Canada, Japan, Australia, and New Zealand [18].

Finally, the respondents were asked about gender, age, the highest completed education, employment status, and the use of headwear (yes/no). The questionnaire was inputted into the SurveyXact (Ramboll) online survey system. The electronic version of the questionnaire was tested with the staff of one of the community pharmacies to assess feasibility of the questionnaire’s outline and time needed to answer all the questions.

### 2.2. Recruitment

Convenience and snowball sampling techniques were used to recruit the study participants. Invitations with a link to the questionnaire were sent to relevant Danish Facebook groups and distributed by emails to the networks of the author, who has a non-Western ethnic background (EO). The recruitment of participants with ethnic minority backgrounds via Facebook groups has been previously used in other Danish studies [19]. Data were collected in January 2020.

### 2.3. Statistical Analyzses

To assess knowledge, attitudes, and behaviors, as well as their associations with age and education, descriptive statistics and bivariate analyses were employed. Parametric and non-parametric tests were used for normally and not normally distributed outcomes, respectively, with histograms used to determine distributions. Medians with interquartile range (IQR) and Mann–Whitney U tests were used for the description and bivariate analyses of not normally distributed outcomes; means with standard deviations (SD), Chi Square, one-way ANOVA, as well as Student’s t tests were used for the description and analysis of normally distributed outcomes. Bonferroni’s test was used to investigate the *p* values for multiple testing; *p* values of less than 0.05 were considered statistically significant. IBM SPSS Statistics version 26 was used for all data analyses.

### 2.4. Ethical Statement

Data were collected in anonymized form, and all subjects checked the informed consent box, provided in the first page of the online questionnaire, before they proceeded participating in the study. According to the Danish law, the Ethical Committee assessment was not needed, as the study did not collect any biological material. 

## 3. Results

In total, 826 people responded to a questionnaire; of these, 254 were women who identified themselves as having a non-Western ethnic background. For the majority of these women, Denmark was the place of birth (162 or 64%); also, for the majority of the women, their mothers (248 or 98%) and fathers (248 or 98%) were born in other places than Denmark, mainly Turkey and Somalia (Supplementary Material 1). The median age (IQR) of these women was 26 (23–33) years old; 81 (32%) had a professional bachelor’s, 71 (28%) had high school, and 56 (22%) had a master’s or a higher university degree. The majority were full-time employed (80 or 32%) and students with work (79 or 31%); 93 (37%) used headwear (Table 1).

The respondents scored highest on the general vitamin D knowledge, with the median (IQR) being 85 (75–90) on the scale of 0 to 100; vitamin D nutrition knowledge, attitudes, and behavior scored lower, but on overage above the middle of the scale (Table 2).

**Figure 1:**
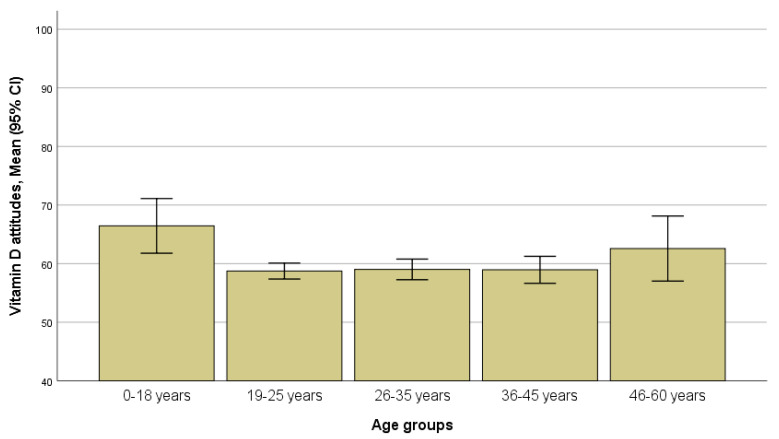
Scores for vitamin D attitudes in age groups (*p* = 0.019, ANOVA).

**Figure 2:**
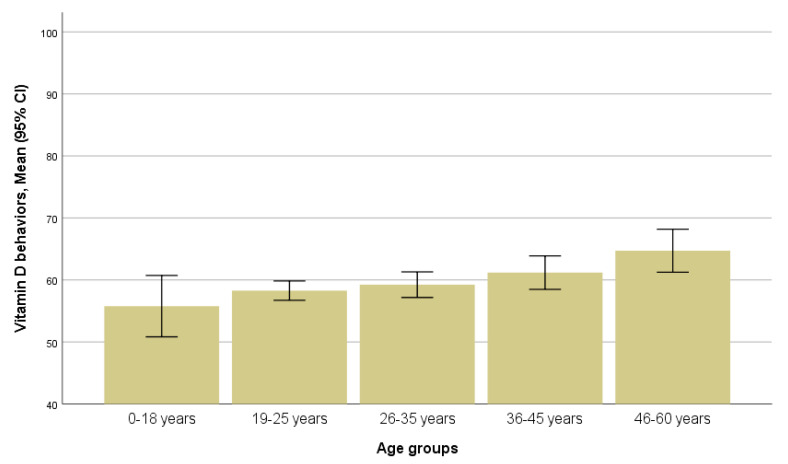
Scores for vitamin D behaviors in age groups (*p* = 0.022, ANOVA).

Women’s age was associated with vitamin D attitudes (*p* = 0.019) and behaviors (*p* = 0.022); younger women scored higher on vitamin D attitudes, and older women scored higher on vitamin D behaviors (Figure 1 and Figure 2). Bonferroni’s test revealed that those in the age groups 19–25 years and 26–35 years had significantly lower scores on vitamin D attitudes than those in the age group of 19–25 years; those in age group 19–25 years had a tendency toward lower scores on vitamin D behaviors than those in the age group of 46–60 years (Supplementary Material 2).

**Figure 3:**
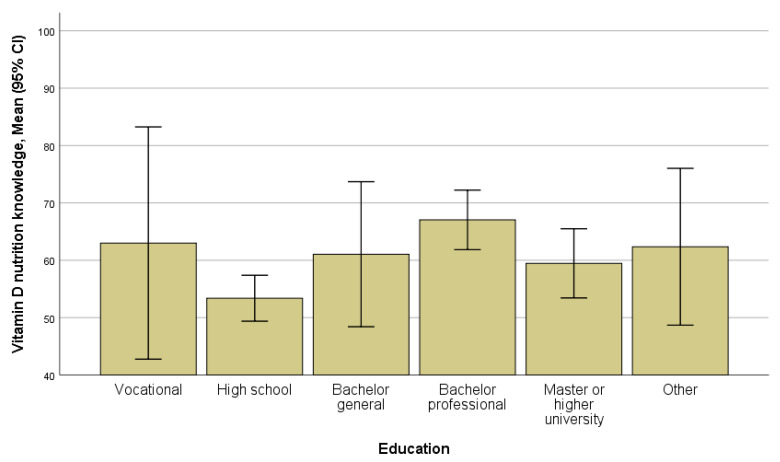
Scores for vitamin D nutrition knowledge in education groups (*p* = 0.014, ANOVA).

Education was associated with vitamin D nutrition knowledge (*p* = 0.014): women with high school education had significantly lower scores on knowledge about vitamin D nutrition compared to those with a professional bachelor’s education (Figure 3, Supplementary Material 3). There were no differences in vitamin D knowledge, attitudes, and behaviors between women with different employment statuses.

## 4. Discussion

The aim of this study was to explore vitamin D knowledge, attitudes, and behaviors in Danish women with non-Western ethnic minority background. The study has found that the general vitamin D knowledge was good, better than vitamin D nutrition knowledge, or vitamin D attitudes and behaviors. Vitamin D nutrition knowledge, attitudes, and behaviors varied depending on women’s education and age. In the following, the characteristics of our respondents, our main findings and methodology of the study will be discussed in more detail.

The majority of women who answered the questionnaire were young and well educated. A large proportion of women had education at bachelor or master level or still were students; many of the women had a full-time job or were both studying and working. This aligns well with the fact that the online version of the questionnaire was distributed on Facebook and via e-mails to the authors’ network. Usually younger individuals spend time in social media and communicate using e-mails. This further suggests that our sample was a selected sample of non-Western ethnic minority women in Denmark, presumably with a higher socioeconomic status as compared to the average socioeconomic status in this population group.

Our study showed a fairly good general knowledge of vitamin D in non-Western ethnic minority women. However, as noted, our respondents were generally young, quite well educated, and studying or already having bachelor’s or master’s degrees. As previous studies showed that higher education is associated with better vitamin D knowledge, usually attained at universities [20,21], with this study we cannot exclude the possibility that non-Western ethnic minority women with low or no education may lack general knowledge about vitamin D and awareness about its importance for health. Interestingly, in our study vitamin D nutrition knowledge, even though on average worse than general knowledge, was best among the respondents with a professional bachelor’s education. Supposedly, some women with a professional bachelor’s education studied nursing or social work and in their studies gained some knowledge on health and/or nutrition. A similar explanation for specific education impact on vitamin D knowledge was provided in a vitamin D knowledge survey in the UK [21]. Notably, our findings on vitamin D knowledge and its association with education resemble the findings from the other studies, not targeted and not specific for ethnic minorities’ populations. As regards the level of vitamin D knowledge in the studies from Western [20,21,22] and non-Western countries [17,23,24], it is hard to compare due to differences in the questions asked. A comparison of the percentages of correct answers (e.g., 80% in Iran [17] and 30% in Canada [22]) in such a context does not make much sense.

In our study, age was a factor determining the vitamin D attitudes and behaviors of the non-Western ethnic women: those younger than 18 years old scored highest on vitamin D attitudes, and those older than 45 years old scored highest on vitamin D behaviors. As the “attitude” concept in the questionnaire we used to represent knowledge on behaviors that could assure vitamin status optimization, and “behavior” concept represented actions to do that, such a finding suggests that older women were more careful in sustaining health and preventing diseases, even though younger ones were more knowledgeable on how one could do that. Probably young women do not perceive themselves at risk, suggesting that knowledge may be not enough to maintain health supporting behaviors. The results concerning the impact of age on vitamin D knowledge, attitudes, and behaviors in the previous studies were not conclusive [20,25].

Our study has a few limitations. First, we did not run a psychometric validation of the Danish translation of the vitamin D knowledge, attitudes, and behaviors’ questionnaire, considering that the pilot test was sufficient for our purposes. The questions on ethnicity and sociodemographic characteristics in the questionnaire were self-constructed. For the sociodemographic questions, usual Danish phrasing and response options were used, and we did not anticipate validity problems here. Ethnicity in the study was defined as self-reported ethnicity, and operationalized via categorization used by Statistics Denmark: Danes, Western immigrants and descendants (i.e., children of immigrants), and non-Western immigrants and descendants. Our initial intention was to categorize ethnicity based on our respondents’ and their mothers’ and fathers’ country of origin, using self-reported ethnicity for sensitivity check. However, a large number of respondents did not provide information on their country of origin, therefore we used self-reported ethnicity for ethnicity categorization. The analyses of those who provided information on the country of origin showed that the majority of the respondents were children of people who immigrated from Turkey and Somalia, which reflected well the Statistics Denmark data [26]. Some individuals, being born and raised in Denmark and talking fluently Danish may consider themselves to be Danes, even if their parents came from abroad, which, according to the Statistics Denmark definition, would make them descendants. Thus, we may have missed some individuals who would be considered as non-Western descendants by Statistics Denmark.

Another limitation of our study is its generalizability: we do not think that the respondents in the study represent all the non-Western ethnic minority women in Denmark. There are a few reasons to claim that. Firstly, the questionnaire was in Danish, so all who do not speak the language were automatically excluded. Our choice to distribute the questionnaire in Danish was based on the intention to reach for diversity of non-Western ethnic minority groups. Secondly, the questionnaire was distributed online, and only those who have e-mails and are members of Facebook groups could be reached. Furthermore, the majority of the questionnaires was distributed to the university students, which was reflected in educational background of the respondents. To raise representability of similar surveys in the future, other ways to reach out and recruit non-Western ethnic minorities (i.e., via nongovernmental organizations, religious or cultural communities) should be explored and utilized. Moreover, as non-Western immigrants and descendants is a culturally heterogeneous group, in the future studies in more homogenous with respect to nationality and religion samples, as well as in depth qualitative studies should be conducted to further explore the specifics of vitamin D knowledge, attitudes, and behaviors in non-Western minorities in the Nordic countries.

## 5. Conclusions

Our study found that general vitamin D knowledge was fairly good among the study participants, who were mainly young well-educated women with a non-Western ethic minority background living in Denmark. In order to better understand vitamin D knowledge, attitudes, and behaviors in non-Western ethnic minority populations in the Nordic countries, in-depth qualitative studies, studies restricted by nationality and religion ethnic minority groups, and studies among those from low socioeconomic strata should be conducted.

## Figures and Tables

**Table 1:** Sociodemographic characteristics of the respondents.

Characteristics	Variables	N	%
Age	0–18 years old	10	4
	19–25 years old	118	46
	26–35 years old	71	28
	36–45 years old	41	16
	46–60 years old	14	5
Education	Vocational	10	4
	High school	71	28
	Bachelor’s general	19	7
	Bachelor’s professional	81	32
	Master’s and higher	56	22
	Other	17	7
Employment	Full time	80	31
	Part time	19	7
	Retired	1	0
	Student	60	24
	Student with work	79	31
	Home wife	8	3
	Other	7	3
Headwear	Yes	93	37
	No	157	62
	Other	4	2
Place of birth	Denmark	162	64
	Other	92	36
Mother’s place of birth	Denmark	6	2
	Other	248	98
Father’s place of birth	Denmark	5	2
	Other	247	97
	Don’t know	1	0

**Table 2:** Scores for vitamin D knowledge, attitudes, and behaviors.

Outcome	N	Median (IQR)	Mean (SD)
Vitamin D general knowledge	254	85 (75–90)	82.8 (12.23)
Vitamin D nutrition knowledge	254	60 (40–80)	60.6 (22.68)
Vitamin D attitudes	243	60 (54–64)	59.4 (7.48)
Vitamin D behaviors	238	60 (54–64)	59.3 (8.27)

## Supplementary Materials

The following are available online at https://www.mdpi.com/1660-4601/17/21/8053/s1: Supplementary Material 1: Country of origin of participants’ mothers and fathers. Supplementary Material 2: Bonferroni test for age groups, Supplementary Material 3: Bonferroni test for education groups.
