# Supplementary material for: Vitamin D Knowledge, Attitudes, and Behaviors in Young Danish Women with a Non-Western Ethnic Minority Background—A Questionnaire Survey
Source: Int J Environ Res Public Health. 2020 Nov 1;17(21):8053. doi: 10.3390/ijerph17218053 (PMC7662621; doi:10.3390/ijerph17218053)
Supplement: Supplementary file 1 [file ijerph-17-08053-s001.pdf]

1. **Country of origin of participants' parents**

Country of mother's origin

Country	Frequency	Percent
Turkey	47	18.5
Somalia	25	9.8
Libanon	8	3.1
Iraq	7	2.8
Afghanistan	6	2.4
Morocco	5	2.0
Pakistan	5	2.0
Iran	3	1.2
Bosnia	2	0.8
Vietnam	2	0.8
Algeria	1	0.4
United Arab Emirates	1	0.4
Kenya	1	0.4
Montenegro	1	0.4
Qatar	1	0.4
Germany	1	0.4
Missing	138	54.3
Total	254	100

Country of father's origin

Country	Frequency	Percent
Turkey	45	17.7
Somalia	25	9.8
Iraq	10	3.9
Lebanon	8	3.1
Afghanistan	6	2.4
Morocco	4	1.6
Pakistan	3	1.2
Bosnia	2	0.8
Iran	2	0.8
Algeria	1	0.4
Burma	1	0.4
Dubai	1	0.4
Egypt	1	0.4
Ethiopia	1	0.4
Kenya	1	0.4
Kina	1	0.4
Macedonia	1	0.4
Montenegro	1	0.4
Uganda	1	0.4
Vietnam	1	0.4
Missing	137	53.9
Total	254	100

2. Multiple Comparisons, Age groups

Bonferroni test

Dependent Variable	Age I	Age J	Mean Difference (I-J)	Std. Error	Sig.	95% Confidence Interval Lower Bound	95% Confidence Interval Upper Bound
General knowledge	0-18	19-25	-5,88983	4,03961	1,000	-17,3311	5,5514
		26-35	-5,59859	4,14275	1,000	-17,3320	6,1348
		36-45	-3,96341	4,32582	1,000	-16,2153	8,2885
		46-60	-6,07143	5,07829	1,000	-20,4545	8,3116
	19-25	0-18	5,88983	4,03961	1,000	-5,5514	17,3311
		26-35	,29124	1,84220	1,000	-4,9264	5,5088
		36-45	1,92642	2,22352	1,000	-4,3712	8,2240
		46-60	-,18160	3,46703	1,000	-10,0012	9,6380
	26-35	0-18	5,59859	4,14275	1,000	-6,1348	17,3320
		19-25	-,29124	1,84220	1,000	-5,5088	4,9264
		36-45	1,63518	2,40582	1,000	-5,1788	8,4491
		46-60	-,47284	3,58667	1,000	-10,6313	9,6856
	36-45	0-18	3,96341	4,32582	1,000	-8,2885	16,2153
		19-25	-1,92642	2,22352	1,000	-8,2240	4,3712
		26-35	-1,63518	2,40582	1,000	-8,4491	5,1788
		46-60	-2,10801	3,79665	1,000	-12,8612	8,6451
	46-60	0-18	6,07143	5,07829	1,000	-8,3116	20,4545
		19-25	,18160	3,46703	1,000	-9,6380	10,0012
		26-35	,47284	3,58667	1,000	-9,6856	10,6313
		36-45	2,10801	3,79665	1,000	-8,6451	12,8612
Nutrition knowledge	0-18	19-25	-4,62712	7,41560	1,000	-25,6301	16,3758
		26-35	-11,92958	7,60494	1,000	-33,4688	9,6096
		36-45	-11,87805	7,94100	1,000	-34,3691	10,6130
		46-60	-4,14286	9,32232	1,000	-30,5461	22,2604
	19-25	0-18	4,62712	7,41560	1,000	-16,3758	25,6301

		26-35	-7,30246	3,38176	,318	-16,8805	2,2756
		36-45	-7,25093	4,08176	,769	-18,8116	4,3097
		46-60	,48426	6,36450	1,000	-17,5417	18,5102
	26-35	0-18	11,92958	7,60494	1,000	-9,6096	33,4688
		19-25	7,30246	3,38176	,318	-2,2756	16,8805
		36-45	,05153	4,41642	1,000	-12,4569	12,5600
		46-60	7,78672	6,58413	1,000	-10,8613	26,4347
	36-45	0-18	11,87805	7,94100	1,000	-10,6130	34,3691
		19-25	7,25093	4,08176	,769	-4,3097	18,8116
		26-35	-,05153	4,41642	1,000	-12,5600	12,4569
		46-60	7,73519	6,96960	1,000	-12,0046	27,4750
	46-60	0-18	4,14286	9,32232	1,000	-22,2604	30,5461
		19-25	-,48426	6,36450	1,000	-18,5102	17,5417
		26-35	-7,78672	6,58413	1,000	-26,4347	10,8613
		36-45	-7,73519	6,96960	1,000	-27,4750	12,0046
Attitudes	0-18	19-25	7,70571*	2,55101	**,028**	,4776	14,9339
		26-35	7,42995*	2,60859	,048	,0387	14,8213
		36-45	7,49444	2,71551	,062	-,1998	15,1887
		46-60	3,87302	3,14473	1,000	-5,0374	12,7834
	19-25	0-18	-7,70571*	2,55101	**,028**	-14,9339	-,4776
		26-35	-,27575	1,12838	1,000	-3,4730	2,9214
		36-45	-,21126	1,35738	1,000	-4,0573	3,6348
		46-60	-3,83269	2,08754	,676	-9,7476	2,0822
	26-35	0-18	-7,42995*	2,60859	**,048**	-14,8213	-,0387
		19-25	,27575	1,12838	1,000	-2,9214	3,4730
		36-45	,06449	1,46273	1,000	-4,0801	4,2090
		46-60	-3,55694	2,15752	1,000	-9,6702	2,5563
	36-45	0-18	-7,49444	2,71551	,062	-15,1887	,1998
		19-25	,21126	1,35738	1,000	-3,6348	4,0573

		26-35	-,06449	1,46273	1,000	-4,2090	4,0801
		46-60	-3,62143	2,28564	1,000	-10,0977	2,8548
	46-60	0-18	-3,87302	3,14473	1,000	-12,7834	5,0374
		19-25	3,83269	2,08754	,676	-2,0822	9,7476
		26-35	3,55694	2,15752	1,000	-2,5563	9,6702
		36-45	3,62143	2,28564	1,000	-2,8548	10,0977
Behavior	0-18	19-25	-2,50000	2,82445	1,000	-10,5045	5,5045
		26-35	-3,45752	2,88765	1,000	-11,6412	4,7261
		36-45	-5,40171	3,01052	,741	-13,9336	3,1302
		46-60	-8,93651	3,47819	,108	-18,7938	,9207
	19-25	0-18	2,50000	2,82445	1,000	-5,5045	10,5045
		26-35	-,95752	1,26027	1,000	-4,5291	2,6141
		36-45	-2,90171	1,52086	,576	-7,2118	1,4084
		46-60	-6,43651	2,31248	**,058**	-12,9901	,1171
	26-35	0-18	3,45752	2,88765	1,000	-4,7261	11,6412
		19-25	,95752	1,26027	1,000	-2,6141	4,5291
		36-45	-1,94419	1,63523	1,000	-6,5785	2,6901
		46-60	-5,47899	2,38926	,227	-12,2502	1,2922
	36-45	0-18	5,40171	3,01052	,741	-3,1302	13,9336
		19-25	2,90171	1,52086	,576	-1,4084	7,2118
		26-35	1,94419	1,63523	1,000	-2,6901	6,5785
		46-60	-3,53480	2,53639	1,000	-10,7230	3,6534
	46-60	0-18	8,93651	3,47819	,108	-,9207	18,7938
		19-25	6,43651	2,31248	**,058**	-,1171	12,9901
		26-35	5,47899	2,38926	,227	-1,2922	12,2502
		36-45	3,53480	2,53639	1,000	-3,6534	10,7230

*. The mean difference is significant at the 0.05 level.

3. Multiple Comparisons, education groups

Bonferroni test

Dependent Variable	Education I	Education J	Mean Difference (I-J)	Std. Error	Sig.	95% Confidence Interval Lower Bound	95% Confidence Interval Upper Bound
General knowledge	Vocational	High school	-7,10563	4,07383	1,000	-19,1801	4,9688
		Bachelor general	-6,86842	4,71207	1,000	-20,8346	7,0977
		Bachelor proffesional	-7,52469	4,04267	,958	-19,5068	4,4574
		Master or higher university	-10,48214	4,14064	,180	-22,7546	1,7903
		Other	-,97059	4,80670	1,000	-15,2172	13,2760
	High school	Vocational	7,10563	4,07383	1,000	-4,9688	19,1801
		Bachelor general	,23721	3,11534	1,000	-8,9964	9,4708
		Bachelor proffesional	-,41906	1,96083	1,000	-6,2308	5,3927
		Master or higher university	-3,37651	2,15560	1,000	-9,7655	3,0125
		Other	6,13505	3,25670	,911	-3,5175	15,7876
	Bachelor general	Vocational	6,86842	4,71207	1,000	-7,0977	20,8346
		High school	-,23721	3,11534	1,000	-9,4708	8,9964
		Bachelor proffesional	-,65627	3,07447	1,000	-9,7687	8,4562
		Master or higher university	-3,61372	3,20221	1,000	-13,1048	5,8773
		Other	5,89783	4,02661	1,000	-6,0367	17,8323
	Bachelor professional	Vocational	7,52469	4,04267	,958	-4,4574	19,5068
		High school	,41906	1,96083	1,000	-5,3927	6,2308
		Bachelor general	,65627	3,07447	1,000	-8,4562	9,7687
		Master or higher university	-2,95745	2,09611	1,000	-9,1701	3,2552
		Other	6,55410	3,21763	,641	-2,9826	16,0908
	Master or higher university	Vocational	10,48214	4,14064	,180	-1,7903	22,7546
		High school	3,37651	2,15560	1,000	-3,0125	9,7655
		Bachelor general	3,61372	3,20221	1,000	-5,8773	13,1048
		Bachelor proffesional	2,95745	2,09611	1,000	-3,2552	9,1701
		Other	9,51155	3,33989	,072	-,3876	19,4107
	other	Vocational	,97059	4,80670	1,000	-13,2760	15,2172
		High school	-6,13505	3,25670	,911	-15,7876	3,5175
		Bachelor general	-5,89783	4,02661	1,000	-17,8323	6,0367
		Bachelor proffesional	-6,55410	3,21763	,641	-16,0908	2,9826
		Master or higher university	-9,51155	3,33989	,072	-19,4107	,3876

Nutrition knowledge	Vocational	High school	9,61972	7,51772	1,000	-12,6621	31,9015
		Bachelor general	1,94737	8,69551	1,000	-23,8253	27,7201
		Bachelor proffesional	-4,03704	7,46021	1,000	-26,1484	18,0743
		Master or higher university	3,53571	7,64101	1,000	-19,1115	26,1830
		Other	,64706	8,87014	1,000	-25,6432	26,9373
	High school	Vocational	-9,61972	7,51772	1,000	-31,9015	12,6621
		Bachelor general	-7,67235	5,74895	1,000	-24,7117	9,3670
		Bachelor proffesional	-13,65676*	3,61845	**,003**	-24,3815	-2,9320
		Master or higher university	-6,08400	3,97788	1,000	-17,8741	5,7061
		Other	-8,97266	6,00981	1,000	-26,7852	8,8399
	Bachelor general	Vocational	-1,94737	8,69551	1,000	-27,7201	23,8253
		High school	7,67235	5,74895	1,000	-9,3670	24,7117
		Bachelor proffesional	-5,98441	5,67353	1,000	-22,8002	10,8314
		Master or higher university	1,58835	5,90926	1,000	-15,9261	19,1028
		Other	-1,30031	7,43058	1,000	-23,3239	20,7232
	Bachelor professional	Vocational	4,03704	7,46021	1,000	-18,0743	26,1484
		High school	13,65676*	3,61845	**,003**	2,9320	24,3815
		Bachelor general	5,98441	5,67353	1,000	-10,8314	22,8002
		Master or higher university	7,57275	3,86809	,771	-3,8919	19,0374
		Other	4,68410	5,93771	1,000	-12,9147	22,2829
	Master or higher university	Vocational	-3,53571	7,64101	1,000	-26,1830	19,1115
		High school	6,08400	3,97788	1,000	-5,7061	17,8741
		Bachelor general	-1,58835	5,90926	1,000	-19,1028	15,9261
		Bachelor proffesional	-7,57275	3,86809	,771	-19,0374	3,8919
		Other	-2,88866	6,16334	1,000	-21,1562	15,3789
	other	Vocational	-,64706	8,87014	1,000	-26,9373	25,6432
		High school	8,97266	6,00981	1,000	-8,8399	26,7852
		Bachelor general	1,30031	7,43058	1,000	-20,7232	23,3239
		Bachelor proffesional	-4,68410	5,93771	1,000	-22,2829	12,9147

		Master or higher university	2,88866	6,16334	1,000	-15,3789	21,1562
Attitudes	Vocational	High school	5,48571	2,49750	,435	-1,9200	12,8914
		Bachelor general	6,95000	2,97808	,307	-1,8808	15,7808
		Bachelor proffesional	6,43077	2,48144	,152	-,9273	13,7889
		Master or higher university	7,08679	2,54708	,087	-,4659	14,6395
		Other	2,95000	2,97808	1,000	-5,8808	11,7808
	High school	Vocational	-5,48571	2,49750	,435	-12,8914	1,9200
		Bachelor general	1,46429	2,04715	1,000	-4,6060	7,5346
		Bachelor proffesional	,94505	1,21631	1,000	-2,6616	4,5517
		Master or higher university	1,60108	1,34516	1,000	-2,3877	5,5898
		Other	-2,53571	2,04715	1,000	-8,6060	3,5346
	Bachelor general	Vocational	-6,95000	2,97808	,307	-15,7808	1,8808
		High school	-1,46429	2,04715	1,000	-7,5346	4,6060
		Bachelor proffesional	-,51923	2,02753	1,000	-6,5314	5,4929
		Master or higher university	,13679	2,10735	1,000	-6,1120	6,3856
		Other	-4,00000	2,61195	1,000	-11,7451	3,7451
	Bachelor professional	Vocational	-6,43077	2,48144	,152	-13,7889	,9273
		High school	-,94505	1,21631	1,000	-4,5517	2,6616
		Bachelor general	,51923	2,02753	1,000	-5,4929	6,5314
		Master or higher university	,65602	1,31510	1,000	-3,2436	4,5556
		Other	-3,48077	2,02753	1,000	-9,4929	2,5314
	Master or higher university	Vocational	-7,08679	2,54708	,087	-14,6395	,4659
		High school	-1,60108	1,34516	1,000	-5,5898	2,3877
		Bachelor general	-,13679	2,10735	1,000	-6,3856	6,1120
		Bachelor proffesional	-,65602	1,31510	1,000	-4,5556	3,2436
		Other	-4,13679	2,10735	,762	-10,3856	2,1120
	other	Vocational	-2,95000	2,97808	1,000	-11,7808	5,8808
		High school	2,53571	2,04715	1,000	-3,5346	8,6060
		Bachelor general	4,00000	2,61195	1,000	-3,7451	11,7451

		Bachelor proffesional	3,48077	2,02753	1,000	-2,5314	9,4929
		Master or higher university	4,13679	2,10735	,762	-2,1120	10,3856
Behaviors	Vocational	High school	2,69412	2,79090	1,000	-5,5834	10,9717
		Bachelor general	-,72500	3,32184	1,000	-10,5773	9,1273
		Bachelor proffesional	,40000	2,77201	1,000	-7,8215	8,6215
		Master or higher university	1,74615	2,84542	1,000	-6,6931	10,1854
		Other	-2,10000	3,32184	1,000	-11,9523	7,7523
	High school	Vocational	-2,69412	2,79090	1,000	-10,9717	5,5834
		Bachelor general	-3,41912	2,28969	1,000	-10,2101	3,3719
		Bachelor proffesional	-2,29412	1,37554	1,000	-6,3738	1,7856
		Master or higher university	-,94796	1,51805	1,000	-5,4504	3,5544
		Other	-4,79412	2,28969	,560	-11,5851	1,9969
	Bachelor general	Vocational	,72500	3,32184	1,000	-9,1273	10,5773
		High school	3,41912	2,28969	1,000	-3,3719	10,2101
		Bachelor proffesional	1,12500	2,26662	1,000	-5,5976	7,8476
		Master or higher university	2,47115	2,35583	1,000	-4,5160	9,4583
		Other	-1,37500	2,91345	1,000	-10,0160	7,2660
	Bachelor professional	Vocational	-,40000	2,77201	1,000	-8,6215	7,8215
		High school	2,29412	1,37554	1,000	-1,7856	6,3738
		Bachelor general	-1,12500	2,26662	1,000	-7,8476	5,5976
		Master or higher university	1,34615	1,48302	1,000	-3,0524	5,7447
		Other	-2,50000	2,26662	1,000	-9,2226	4,2226
	Master or higher university	Vocational	-1,74615	2,84542	1,000	-10,1854	6,6931
		High school	,94796	1,51805	1,000	-3,5544	5,4504
		Bachelor general	-2,47115	2,35583	1,000	-9,4583	4,5160
		Bachelor proffesional	-1,34615	1,48302	1,000	-5,7447	3,0524
		Other	-3,84615	2,35583	1,000	-10,8333	3,1410
	other	Vocational	2,10000	3,32184	1,000	-7,7523	11,9523
		High school	4,79412	2,28969	,560	-1,9969	11,5851

	Bachelor general	1,37500	2,91345	1,000	-7,2660	10,0160
	Bachelor proffesional	2,50000	2,26662	1,000	-4,2226	9,2226
	Master or higher university	3,84615	2,35583	1,000	-3,1410	10,8333

*. The mean difference is significant at the 0.05 level.
